# Primary Pleural Diffuse Large B-cell non-Hodgkin’s Lymphoma Diagnosed via [18F]-2-Fluoro-Deoxy-DGlucose Positron Emission Tomography /Computed Tomography

**DOI:** 10.5505/tjh.2012.38233

**Published:** 2012-03-05

**Authors:** İlknur Ak Sivrikoz, Zafer Gülbaş

**Affiliations:** 1 Eskişehir Osmangazi University, School of Medicine, Department of Nuclear Medicine, Eskişehir, Turkey; 2 Eskişehir Osmangazi University, School of Medicine, Department of Hematology, Eskişehir, Turkey

Primary pleural lymphoma is a rare entity. We report a64-year-old-man with primary malignant lymphoma arisingin the pleura with no history of persistent pyothorax.Chest computed tomography scan (CT) showed left pleuraleffusion with thickening of the parietal pleura. Therewere no intrapulmonary or mediastinal abnormalities.Analysis of pleural effusion did not detect empyema, tuberculosis,mycobacterium species, or mycelium. Initially,malignant mesothelioma was suspected, but it could notbe diagnosed by cytological examination of pleural fluid.Flow cytometric analysis of pleural fluid showed cytomorphologicand immunophenotypic evidence of diffuse Bcell Non Hodgkin’s Lymphoma (NHL) (Figure 1). [18F]-2-fluoro-deoxy-D-glucose (F-18 FDG) positron emissiontomography/computed tomography (PET/CT) scan revealeda diffuse F-18 FDG uptake on thickened costal anddiaphragmatic parietal pleura in left hemithotax indicatingpleural involvement (Figure 2).Pathological and immunohistochemical(with CEA, LCA, CD20, CD3) examinationof the pleural lesion obtained by pleural biopsy revealedthat it was B-cell of the diffuse large cell type of NHL arising from the pleura . We have written informed consentand no conflict of interest.

Malignant lymphoma arising in the pleura are rare,comprising 2.4% of the primary chest wall tumors, andmost pleural lymphomas develop in association with precedinglong-standing pleural disease such as long-standingchronic tuberculous pyothorax or artificial pneumothoraxfor lung tuberculosis. As a mechanism for pleurallymphoma, it had been suspected that there was chronicstimulation of B-cells in the pleural cavity such as that inlong-standing chronic pleural disease, because it was reportedthat the most common malignant lymphoma arisingin the pleura was B-cell non-Hodgkin’s lymphomaof the diffuse large cell type histologically [1,2,3,4,5,6,7]. Humanherpesvirus type 8 (HHV8), also known as Kaposi’s sarcoma-associated herpesvirus, is a human gamma herpesvirusthat underlies the pathogenesis of Kaposi’s sarcoma,primary effusion lymphoma and multicentric Castleman’sdisease. Therefore, Kaposi Sarcoma and Multicentric Castleman’sDisease should be considered in the differentialdiagnosis [8].

**Conflict of Interest Statement**

The authors of this paper have no conflicts of interest,including specific financial interests, relationships, and/or affiliations relevant to the subject matter or materialsincluded.

## Figures and Tables

**Figure 1:**
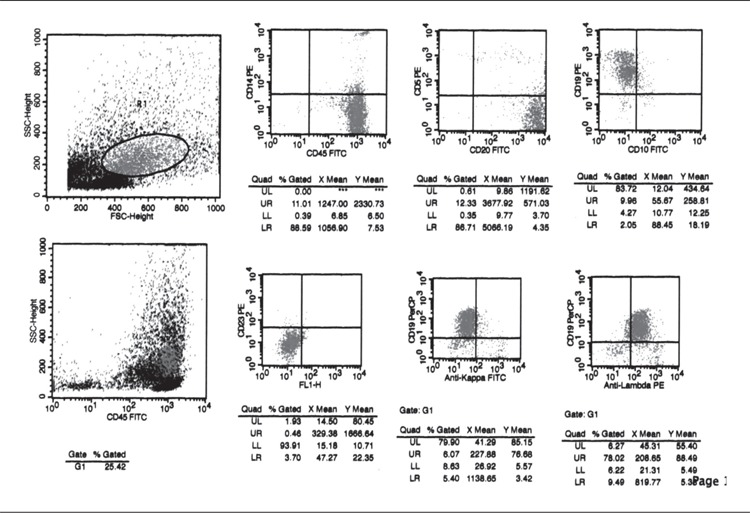
Flow cytometricanalysis of pleural fluidconfirmed the B-cell non-Hodgkin’s lymphoma. CD20positive and CD19 positive cellsare 87% and %84 of the cells,respectively. Anti kappa + CD19expression is negative. Antilambda + CD19 positive cells are78% of the cells.

**Figure 2:**
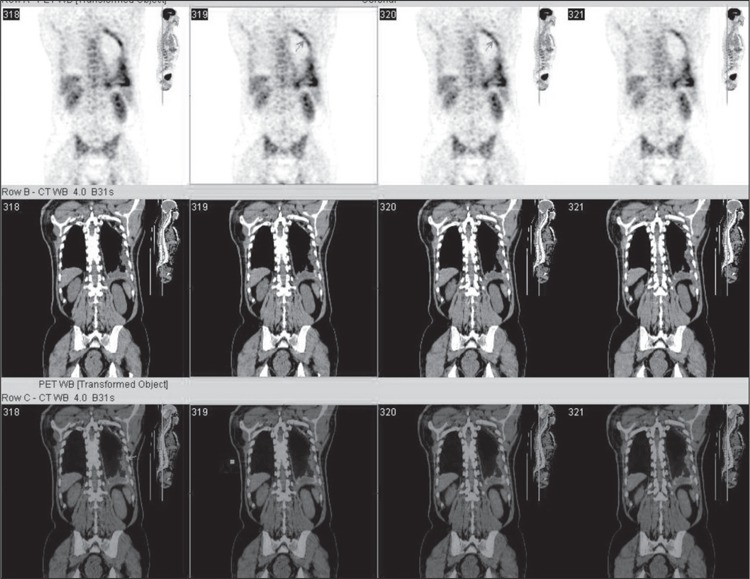
The patient wasimaged using an integratedPET/CT camera (1hour afterthe administration of 465 MBqFDG), which is consists of a6-slice CT gantry integratedon a LSO based full ring PETscanner (Siemens Biograph 6,IL, Chicago, USA). MIP PET,CT and fusion PET/CT imagesshow a diffuse F-18 FDG uptakewith a maximum standarduptake value (SUVmax) of4.2 on thickened mediastinal,costal and diaphragmatic pleurain left hemithotax indicatingpleural involvement. There isno additional focus suggestinglymphomatous disease.
